# Sex bias in lymphocytes: Implications for autoimmune diseases

**DOI:** 10.3389/fimmu.2022.945762

**Published:** 2022-11-24

**Authors:** Katherine C. Dodd, Madhvi Menon

**Affiliations:** ^1^ Lydia Becker Institute of Immunology and Inflammation, Division of Immunology, Immunity to Infection and Respiratory Medicine, Faculty of Biology, Medicine and Health, University of Manchester, Manchester Academic Health Science Centre, Manchester, United Kingdom; ^2^ Manchester Centre for Clinical Neurosciences, Salford Royal Hospital, Salford, United Kingdom

**Keywords:** lymphocytes, sex, sex-bias, T cells, B cells, estrogen, testosterone

## Abstract

Autoimmune diseases are characterized by a significant sex dimorphism, with women showing increased susceptibility to disease. This is, at least in part, due to sex-dependent differences in the immune system that are influenced by the complex interplay between sex hormones and sex chromosomes, with contribution from sociological factors, diet and gut microbiota. Sex differences are evident in the number and function of lymphocyte populations. Women mount a stronger pro-inflammatory response than males, with increased lymphocyte proliferation, activation and pro-inflammatory cytokine production, whereas men display expanded regulatory cell subsets. Ageing alters the immune landscape of men and women in differing ways, resulting in changes in autoimmune disease susceptibility. Here we review the current literature on sex differences in lymphocyte function, the factors that influence this, and the implications for autoimmune disease. We propose that improved understanding of sex bias in lymphocyte function can provide sex-specific tailoring of treatment strategies for better management of autoimmune diseases.

## Background

Autoimmune diseases occur in those who are genetically susceptible, with contributions from environmental and biological factors, resulting in subsequent gene-environment interactions (1). There is wide disparity in the epidemiology of autoimmune diseases between males and females, with variation seen in prevalence, age of onset, phenotype, disease severity, and response to treatment (**Table 1**) (2–4, 8, 11–13, 16). Women mount stronger innate and adaptive immune responses than men, which provides a survival advantage to infectious diseases (32), but also a four times increased likelihood of developing autoimmunity (33, 34). Despite this, sex differences are often not fully investigated or acknowledged in immunological research and treatment strategies. Animal models of autoimmune diseases have historically been predominantly male, resulting in male-biased data influencing treatment decisions, and dual-sex immunology studies frequently failing to explore sex differences (35). More recently, there has been a drive to better understand sex differences in the immune response and distinguish between the effects of hormones and genetic factors. This has resulted in the identification of numerous sex differences in both the innate and adaptive arms of the immune system.

**Table 1:** Sex bias in autoimmune diseases.

Disease	Male: Female	Average age of onset	Disease severity	Effect of pregnancy on disease	References
**Higher prevalence in women**					
**Autoimmune thyroid disease**	1: 4-9	F: 41 **M: 45**	M > **F**	Improves	(2, 3)
**Rheumatoid Arthritis**	1: 2-3	F: 61 **M: 62**	M < **F**	Improves	(4–7)
**Multiple Sclerosis**	1: 2-3	F: 45 **M: 46**	**M** > F	Fewer relapses	(8–10)
**Inflammatory Bowel Disease**	1: 1	**F: 27** **M: 27**	**M** > F	No change in relapse rate	(11–13)
**Coeliac Disease**	1: 12	F: 44.5 **M 48.9**	M < **F**	No change	(14, 15)
**Systemic Lupus Erythematosus**	1: 8	F: 49 **M: 58**	**M** > F	Worsens	(16–18)
**Myasthenia Gravis**	Early-onset: 1: 3 Late-onset: 1.5: 1	F: 51.9 **M: 61.3**	M < **F**	Improves	(19, 20)
**Higher prevalence in men**					
**Psoriasis/Psoriatic arthritis**	1.4: 1	F: 28.7 **M: 32.2**	**M** > F	Improves	(14, 21–23)
**Type 1 Diabetes Mellitus**	1.2: 1	**F: 6.3** M: 6.1	M < **F**	Worsens complications	(24, 25)
**Ankylosing Spondylitis**	1.1: 1	**F: 29.5** M: 27.4	M < **F**	Worsens	(26–28)
**Myocarditis**	3.3: 1	**F: 49.0** M: 34.1	**M** > F	Increases risk	(29–31)

Bold values in “average age of onset” is the older age of the two. Bold in “disease severity” is the gender with greatest severity.

Lymphocytes play a central role in the development and progression of autoimmune diseases. Defects in immune tolerance mechanisms result in the expansion of autoreactive T cells and autoantibody-producing B cells leading to target organ damage and dysfunction (36). Hundreds of genes are differentially expressed between male and female lymphocytes (37). The X chromosome is significantly bigger and more complex than the Y chromosome, and contains many genes relating to immune function, including toll-like receptors (TLRs), cytokine receptors, and transcription factors (38, 39). During embryogenesis, X chromosome inactivation (XCI) randomly silences one of the X chromosomes in females. Around 15% of genes escape XCI in women, particularly those on the short arm which is evolutionarily a more recent addition, and are therefore expressed bi-allelically (40, 41). Incomplete or skewed XCI results in over-expression of X immune-related genes and micro RNAs (mRNA) in female lymphocytes, and is associated with autoimmune diseases (41–47).

Furthermore, lymphocytes express cell membrane and nuclear sex hormone receptors, signaling through which can cause epigenetic and transcriptional changes, thereby regulating the function of the immune system (48, 49). Nuclear hormone receptors, upon engagement, can upregulate the expression of several key immune related genes either directly by binding to hormone response elements in the promoter region, or indirectly by binding to transcription factors which then bind to the gene promoters (49–51). Sex hormone receptors on the cell membrane can activate cytoplasmic signaling and cause signal transduction effects *via* mechanisms such as rapid calcium fluxes (49–51).

The full extent and implication of the sex differences in lymphocytes are yet to be fully elucidated. It is essential to continue to better understand and acknowledge these differences, in order to advance the knowledge of autoimmune disease pathophysiology, and to allow the development of tailored sex-specific therapies. This review focuses on sex differences in lymphocytes, and the implications this has for autoimmune disease pathophysiology.

## Sex bias in T cells

### Development and maturation

Sex-dependent differences in the frequencies of T cells have been reported by multiple independent studies. Females have been shown to display higher frequencies of naïve T cells, cluster of differentiation (CD)4 T cells and higher CD4:CD8 T cell ratios compared to males of the same age (52–55). Conversely, males display higher frequencies of CD8 T cells, and a higher CD8:CD4 T cell ratio (52–55). These differences in T cell subset frequencies have been shown to be influenced by sex hormones (51, 55). For instance, castration of male mice results in thymic enlargement, increased output of naïve, CD4 and CD8 T cells, and increase in autoimmune disease susceptibility, as observed in female mice, indicating that testosterone has a suppressive effect on lymphopoiesis (56, 57). These effects of androgens on T cells are observed largely in early development, as T cells lose expression of androgen receptors (ARs) upon maturation. In contrast, estrogen receptors (ERs) continue to be expressed by T cells at later stages of development (58). The effects of estrogen on T cell populations are dose dependent. Whereas low doses have been shown to expand CD4 T cells (59), high doses (observed during pregnancy) reduce CD4 and CD8 T cell numbers (60).

### Proliferation, differentiation and function

T cells can be classified into functionally distinct subsets based on the cytokines they produce. These subsets include interferon (IFN)-γ/interleukin (IL)-2/tumor necrosis factor (TNF)-α-producing T helper 1 (Th1) cells, IL-4/IL-5/IL-13-producing Th2 cells, IL-17/IL-22-producing Th17 cells and regulatory T cells (Tregs) (61). Oestrogen receptors (ER) have two main subtypes, -α and -β, with differing effects upon the T cell milieu. Activation of ER-α, but not ER-β, results in thymic atrophy, drives enhanced T cell activation and proliferation, and alters T cell subset frequencies (55, 60, 62, 63). In contrast, ER-β promotes apoptosis *via* inhibition of B cell lymphoma (Bcl)-2 signaling and inhibits inflammatory genes such as IFN-γ (64, 65). ER-α and -β expression also differs between T cell subsets; both higher in CD4 than CD8 T cells (60, 66).

**Figure 1:**
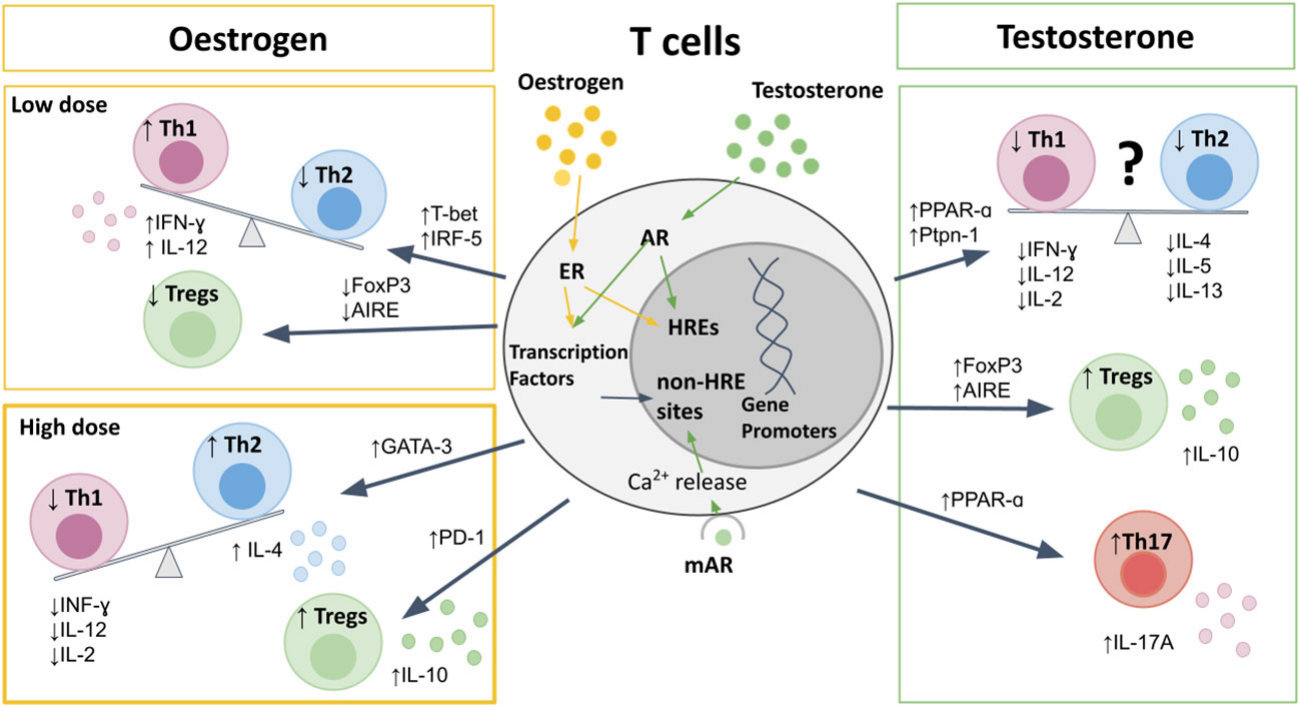
The effects of sex hormones on T cells. Sex hormones bind to nuclear receptors, leading to direct binding to HREs, or indirect effects on gene promotors through transcription factors. Oestrogen at pre-ovulatory concentrations upregulates T-bet and IRF5 to cause a Th1 shift, and downregulates AIRE and FoxP3 to reduce Tregs. High-dose estrogen during pregnancy leads to a Th2 shift, *via* GATA-3, and promotes Treg expression and PD-1 expression. Testosterone upregulates PPAR-α, and Ptpn-1 leading to reduced Th1 responses, but also suppresses Th2 cytokine production, and therefore the Th1/Th2 bias is less clear. Testosterone does increase Th17 production, as well as increasing expression of FoxP3 and AIRE to promote Treg differentiation and increased IL-10 production. AIRE, autoimmune regulator; AR, androgen receptor; ER, estrogen receptor; FoxP3, Forkhead-box-P3; HREs, hormone response elements; IFN, interferon; IL, interleukin; IRF, interferon regulatory factor; mAR, membrane androgen receptor; PPAR, proliferator-activated receptor; Ptpn, protein-tyrosine phosphatase; Th, T-helper; Treg, regulatory T cell.

The Th1/Th2 balance in women shifts with hormonal changes observed during the menstrual cycle and pregnancy (**Figure 1**). Low concentrations of estrogen seen in pre-ovulatory females leads to upregulation of the IFN-γ promoter (67), interferon regulatory factor 5 (IRF5) (68, 69), TLRs (70), and T-bet (60, 71, 72). This promotes an expansion of antigen-specific CD4 T cell responses and a Th1 shift, with increased production of IFN-γ (60, 71, 73). In contrast, during the luteal phase of the menstrual cycle, when estrogen levels increase, a Th2 shift is seen, with increased IL-4 and IL-10 and decreased IFN-γ production (74, 75). This Th2 shift has been shown to be *via* ER-α driven increase in GATA-3 expression (76). Very high levels of estrogen and progesterone that occur in pregnancy further promote Th2 immunity, in order to promote fetal tolerance, with increased production of IL-10 and reduced IL-2, IL-12, and IFN-γ (77–81). The mechanism of these dose-dependent effects remain to be elucidated.

There is conflicting data as to the direction of any Th1/Th2 bias in males when compared to females (82–84). On one hand, some studies have described a predominantly Th1 response in males, with a more pronounced Th2 response in females (82, 85). When comparing Th1 and Th2 cytokines from 20 women and 15 men, Escobar and colleagues reported a higher production of IFN-γ and IL-2, mirrored by reduced IL-10 and IL-4 in men compared to women (82). On the other hand, several other studies in humans and mice have demonstrated decreased frequencies of Th1 in males compared to females (60, 84, 86–89). Moreover, testosterone has been shown to upregulate the expression of peroxisome proliferator-activated receptor (PPAR)-α (90), and protein-tyrosine phosphatase (Ptpn)-1 (88, 91), both of which inhibit Th1 differentiation. This is further supported by studies showing that castration of mice results in upregulation of IFN-γ and T-bet expression (91, 92). The contrasting data may be due to variability between species and different disease settings (89, 93), as well as partly due to the dose-dependent effects of estrogen on the Th1/Th2 balance resulting in a constantly shifting bias between men and women. To further complicate the picture, androgens have also been shown to reduce Th2 cytokines IL-4, IL-5, and IL-13 (94–97), and consistently enhance production of IL-10 from T cells (86, 88, 98, 99).

Although upregulation of PPAR-α expression by androgens suppresses Th1 responses, this has been shown to promote Th17 responses in males compared to females (83, 100). The effects of estrogen on Th17 responses are less clear, likely due to differences in estrogen receptor (ER-α *vs.* ER-β) signaling on Th17 differentiation and function. ER-α signaling has been shown to promote Th17 responses, as ER-α deletion inhibits Th17 differentiation in a mouse model of colitis (101). Moreover, ER-α signaling has been shown to increase production of IL-17A by Th17 cells by promoting mitochondrial respiration, proliferation and by upregulating IL-23R expression (102). In contrast, ER-β signaling in CD4 T cells has been shown to suppress Th17 cell differentiation, as well as promote the induction and maintenance of Tregs (103, 104). Progesterone also suppresses Th17 differentiation, resulting in reduced Th17 responses during pregnancy (4).

Men display higher frequencies of Tregs compared to women due to both genetic and hormonal influences (105). Regulatory protein forkhead box P3 (FoxP3) expression has been shown to be higher in males due to parental imprinting effects, with less methylation on the maternal X allele, which is expressed exclusively in males (106). Additionally, a recent study has demonstrated that Treg frequencies are one of the top features of immune cell profiling that differentiate young post-pubertal men from women, and that male Tregs have higher suppressive capabilities due to increased phosphoinositide 3-kinases (PI3K) signaling (107). The autoimmune regulator (AIRE) gene, which is critical for Treg development, is downregulated by estrogen and progesterone and upregulated by testosterone, thereby resulting in increased thymic expression of AIRE in males compared to females (108). Furthermore, androgens also promote increased expression of the FoxP3 (109), whereas ER-α signaling downregulates FoxP3 expression (101).

Treg numbers fluctuate during the menstrual cycle with maximum expansion in the late follicular phase when estrogen levels are high and the woman is most fertile (110), in order to promote tolerance for a potential pregnancy (110). During pregnancy high levels of estrogen and progesterone further promote Treg differentiation and upregulation of the immunosuppressive molecule programmed cell death protein (PD)-1 (111–117).

## Sex bias in B cells

### Development and maturation

Females display higher frequencies of B cells than males (55), along with enhanced B cell survival, maturation and class switching (118–121). Although both androgens and estrogens can suppress B cell lymphopoiesis (122), only testosterone reduces numbers of B cells in the bone marrow (56, 58). Estrogen promotes hematopoiesis within the liver, which may lead to autoreactive lymphocytes which escape central tolerance mechanisms (123). Oestrogen also interferes with negative selection, resulting in increased survival and expansion of high-affinity autoreactive B cells (118, 124, 125). Studies using ER-α and ER-β deficient mice have shown that engagement of either receptor can affect B cell maturation, but only ER-α engagement can drive an increase in autoreactive B cells (126). In contrast to T cells, B cells express more ER-β than -α (127), however ER-α expression is around four fold higher in B cells than T cells, with higher expression in females compared to males (128), suggesting another mechanism by which females are prone to autoimmunity.

### Proliferation, differentiation and function

**Figure 2:**
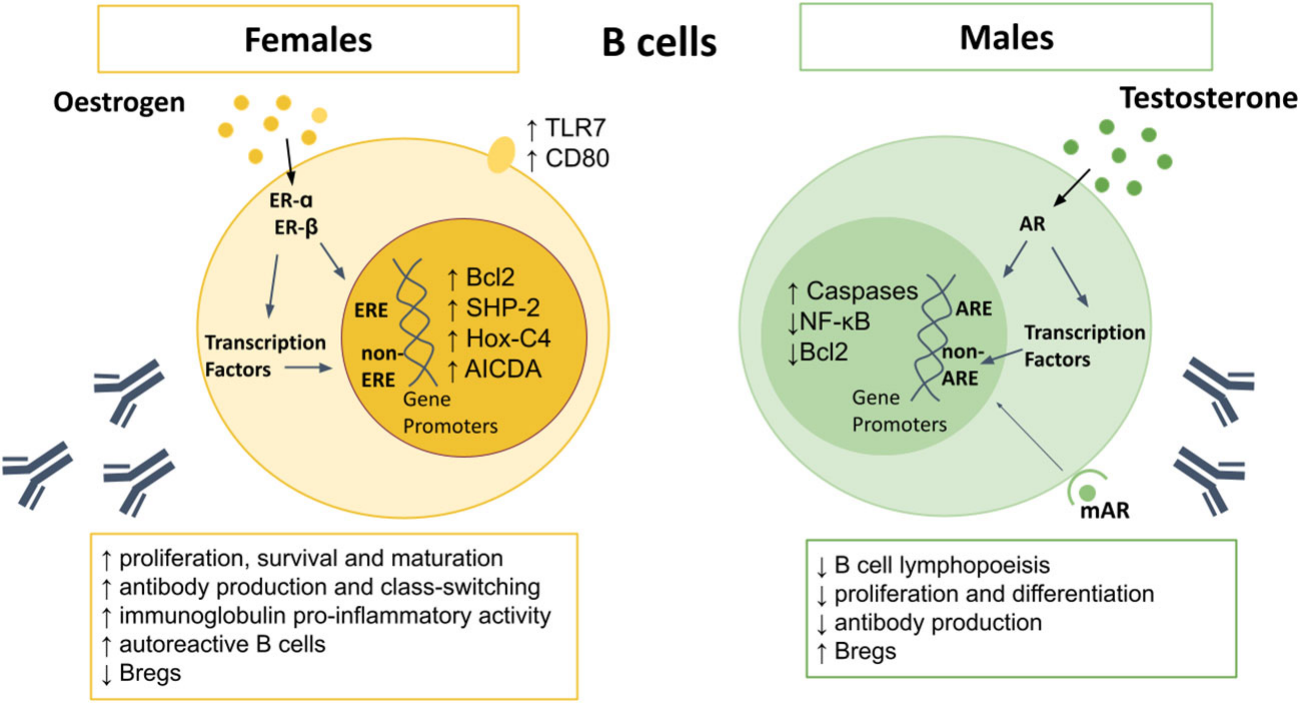
Sex differences in B cells. AR signaling appears to upregulate caspases, and downregulate NF-κB and Bcl2, resulting in increased B cell apoptosis. Males also demonstrate reduced B cell lymphopoiesis in the bone marrow, reduced proliferation and differentiation of B cells, and lower antibody production, but higher Bregs. Oestrogen upregulates Bcl-2, SHP-2, Hox-C4, and AICDA, as well as surface expression of TLR7 and CD80. Female B cells demonstrate higher proliferation, survival and maturation, higher pro-inflammatory antibody production, leading to higher levels of autoreactive B cells. AICDA, Activation-induced cytidine deaminase; AR, androgen receptor; Bcl-2, B cell lymphoma 2; Bregs, regulatory B cells; Hox-C4, homeobox protein C4; IFN, interferon; IL, interleukin; NF-κB, nuclear factor kappa-light-chain-enhancer of activated B cells; SHP-2, Src homology region 2 domain- containing protein tyrosine phosphatase; TLR, toll-like receptor.

Testosterone inhibits proliferation and differentiation of B lymphocytes by reducing B-cell activating factor (BAFF) production by macrophages and through altering key modulators of apoptosis including downregulation of B cell lymphoma (Bcl)-2 and nuclear factor kappa-light-chain-enhancer of activated B cells (NFκB) (**Figure 2**) (49, 129, 130). Conversely, estrogen enhances B cell proliferation, suppresses apoptosis, and promotes survival of autoreactive B cells *via* increased expression of survival regulators BAFF, Bcl-2, CD22, and SH2 containing protein tyrosine phosphatase (SHP)-2 (118, 122, 131–133). Oestrogen also increases expression of CD80, a co-stimulatory signal important for B cell activation (71). In mouse models of arthritis, higher frequencies of IL-10-producing regulatory B cells (Bregs) that exhibit immune suppression have been reported in males compared to females (134). However, the sex bias in Breg frequency and function between men and women in health requires further study.

### Immunoglobulin production

Greater antibody responses and higher basal immunoglobulin (Ig) levels are seen in women (55, 135). Circulating levels of IgM, and in some studies IgG, are higher in females than males (136–138), and are even higher in women with three X chromosomes (139). Higher expression of the X chromosome encoded gene TLR7 in females results in increased IgG class-switching and higher TLR7-driven plasma cell proliferation (140, 141). Oestrogen promotes differentiation of B cells into antibody-secreting cells, with highest frequencies observed just before ovulation, when estrogen is at its peak (142). This is likely an evolutionary attempt to reduce the risk of infection and maximize the chance of conception. Testosterone has the opposite effects and lowers antibody production (122, 135, 143). Moreover, mice with AR-deficient B cells display increased autoantibody production (130).

Activation-induced cytidine deaminase (AICDA) is required for somatic hypermutation and class switch recombination, mechanisms which are important for Ig gene modification in B-cells and antibody production (144). AICDA is upregulated in B-cells by estrogen, due to increased expression of homeobox protein (Hox)-C4, a transcriptional regulator of AICDA (122, 145), explaining the higher antibody production in females to males. However, during pregnancy, progesterone inhibits AICDA transcription, with subsequent reduction in somatic hypermutation and class switch recombination (146), resulting in reduced antibody production.

## Sex bias in lymphocytes in autoimmune diseases

It is important to consider the implications of sex differences in lymphocytes within the context of specific autoimmune diseases. Variation in lymphocyte responses between males and females influence disease susceptibility and severity in differing ways dependent upon disease mechanism. Although less common, some autoimmune diseases have a slight male bias, such as late onset myasthenia gravis (LOMG) (1.5:1), ankylosing spondylitis (1.1:1), myocarditis (3.3:1) and type 1 diabetes mellitus (1.2:1) (see **Table 1**) (14, 21, 24, 25). Males who do develop female predominant autoimmune diseases, such as rheumatoid arthritis (RA) and systemic lupus erythematosus (SLE), have lower androgen concentrations than controls, which likely skews their lymphocyte profile towards autoimmunity (147–149). Additionally, older men are at higher risk of loss of central tolerance mechanisms associated with thymic involution, as seen in LOMG (150). Here we compare and contrast sex differences identified in four common autoimmune diseases; SLE, multiple sclerosis (MS), RA, and myasthenia gravis (MG).

### Systemic lupus erythematosus

SLE is a systemic autoimmune condition that can affect multiple organs and tissues and is characterized by autoantibodies directed towards a wide spectrum of cellular components, most commonly anti-nuclear antibody (151). It is a highly heterogeneous disease that can cause symptoms ranging from malar rash and fever to arthritis and nephritis (152).

The expression of miRNAs and immune related genes associated with SLE are frequently higher in females, due to effects of skewed XCI, partial reactivation, DNA demethylation, and estrogen binding (153–156), contributing to the female predominance of the disease. Several upregulated miRNAs target CBL, which increases T cell activation and accelerates T cell activation-induced cell death (153–156). Mature naïve T and B lymphocytes from females with SLE display higher expression of CXCR3, which drives Th1 and Th17 infiltration into the kidney in lupus nephritis (157, 158), and CD40LG, which promotes IgG production (43, 159) (160). TLR7 and CXorf21 (also termed TASL; TLR adapter interacting with SLC15A4 on the lysosome) also escape XCI (140, 141, 161–163), resulting in higher production of IFN-α, which is critical in driving the pathogenesis of SLE (164, 165).

Additionally, different types of lupus nephritis appear to be driven by differing cytokine profiles. Whereas Th1-mediated inflammation promotes diffuse lupus nephritis, seen more commonly in males, Th2 responses result in membranous lupus nephritis, which is more common in females (166, 167). Skin lesions in lupus, also more common in females, are triggered by Th2 cells, which subsequently transition to a Th1-like phenotype as a result of increased TLR7 signaling (168, 169). Therefore, it may be beneficial to target Th1 responses in males and TLR7 in females with SLE.

Oestrogen has been shown to accelerate disease in mouse models of lupus, by driving Th1 and Th2 responses *via* ER-α signaling (63, 170–172). Binding of ER-α also leads to upregulation of TLR8 and IRF5, amplifies production of IFN-α/β, and promotes survival, expansion and activation of autoreactive B cells in SLE (63, 68, 171, 173, 174). The increased production of IFN-α in females has been shown to increase ER-α expression on murine splenic cells, resulting in a positive feedback loop that promotes inflammation (128). ER-α and -β engagement results in a dose-dependent increase in activation of T cells from SLE patients, with increased calcineurin and CD40L expression (175, 176). Overall, it appears that ER-β has an immunosuppressive effect on disease (63). This is further supported by a study demonstrating that patients with SLE have lower ER-β expression, but similar ER-α, in T cells compared to healthy controls (177).

Whereas estrogen has been shown to accelerate disease, androgens administered to mouse models of lupus have been shown to ameliorate disease (174, 178, 179). There are low levels of androgen and progesterone in bodily fluids of SLE patients compared to controls, and higher levels of estradiol, possibly due to increased conversion of androgen precursors to estrogen (147, 148). SLE-related immune complex-mediated glomerulonephritis has been linked to a switch from predominantly IgM anti-ds DNA, to IgG2a and IgG2b, which occurs earlier in disease in females than males and can be delayed by androgen administration (180, 181).

Sex hormone signaling appears to be dysregulated in SLE. In contrast to promoting Treg differentiation in healthy females, estrogen exposure *in vitro* inhibits Treg differentiation in peripheral blood mononuclear cells (PBMCs) from SLE patients (148). This may explain the worsening of disease that is seen with pregnancy in SLE, in contrast to improvements in most other autoimmune diseases during pregnancy (17). This reduction in Treg frequencies seen in SLE patients has been linked to the increased risk of pregnancy-related morbidities, including fetal loss, growth restriction, preterm birth, and pre-eclampsia (182).

Taken together, these studies suggest that chromosomal effects resulting in higher expression of SLE-related genes and miRNAs, in combination with dysregulated ER-α signaling, promote T cell activation and autoantibody production, alongside Treg inhibition. While further studies are required to better understand mechanisms of sex differences in SLE, there is sufficient evidence to conclude that alterations in lymphocyte function contribute to the profound female sex bias in SLE.

### Multiple sclerosis

MS causes a wide range of neurological symptoms, and potentially significant disability, due to inflammation and demyelination of white matter in the central nervous system (CNS) (9). Although women are more likely to develop MS, men are at a higher risk of developing more severe disease (183). MS and its mouse model experimental autoimmune encephalitis (EAE) are considered to be myelin protein-specific Th1- and Th17-mediated diseases (184, 185). Sex differences in cytokine production have been demonstrated in EAE, with greater Th1 activation seen in females, and increased Th17 responses in males (83, 186, 187).

Kdm6a is a histone demethylase on the X chromosome that regulates transcription of numerous genes. It has been identified as one of the top differentially expressed genes on CD4 T cells between males and females, with higher expression in females due to escape from XCI (107, 188). Mice lacking Kdm6a in CD4 T cells have been shown to display a downregulation of neuroinflammation, TLR signaling and IL-17 signaling genes, and are protected from EAE (188), suggesting that this is an important mechanism to explain the female sex bias in MS.

Both estrogen and testosterone display protective effects in MS, but *via* differing mechanisms. Androgen administration to EAE mice has been demonstrated to protect against disease through inducing a Th2 bias (86, 87, 189), which is dependent upon AIRE (108). Unlike what is observed in health, estrogen administration in EAE inhibits Th1 and Th17 responses *via* ER-α binding to suppress disease (103, 104, 190). Oestrogen also promotes B cell migration away from the target organ by upregulating the expression of chemokine CXCL13 (191), which acts through its receptor CXCR5 (widely expressed on mature B cells (192)). Further beneficial effects of estrogen in MS include expansion of PD-L1-expressing Bregs as well as upregulation of PD-1 on Tregs (117, 191), which leads to further downregulation of Th17 responses (117, 191, 193, 194).

Women with MS display a reduction in the incidence of relapses during pregnancy, followed by an increased risk of relapse post-partum (195). In the third trimester, high levels of estrogen modulate CD4 T cell function by promoting a decrease in Th17 cells mirrored by an expansion of Tregs (196). This protective effect is further enhanced by ER-α signaling in Bregs, potentiating Treg activity (197, 198). Additionally, progesterone has also been shown to suppress EAE by promoting IL-10 production by B cells (144, 198). In the post-partum period, B cells from MS patients preferentially develop into plasma cells (199). Furthermore, activated memory T cells from relapsing MS patients produce more pro-inflammatory cytokines when compared to non-relapsing patients and healthy controls postpartum (200).

To summarize, MS is more common in females that males, due to genetic effects such as increased expression of Kdm6a. However, in contrast to its role in SLE, estrogen has immunosuppressive effects on T and B lymphocytes and ameliorates disease.

### Rheumatoid arthritis

RA is a chronic inflammatory disease that results in pain, swelling, stiffness and deformity of multiple joints (5). RA is around three times more common in females than males and has a peak age of onset around the time of menopause in women, but occurs towards later life in men (6). The onset of RA is associated with the post-menopausal and postpartum periods, suggesting links with falling levels of female sex hormones (6, 10, 201). Of note, men with RA have lower androgen concentrations and higher estrogen levels than controls (149).

Nearly all human autoimmune diseases have been linked to specific human leukocyte antigen (HLA) genes, mainly haplotypes in major histocompatibility complex (MHC) class II (202). It is suggested that these haplotypes predispose to autoimmunity by presenting many peptides, with specific haplotypes linked to differing cytokine profiles (203). HLA-DR4 is associated with RA and supports a female sex bias (134, 204). Female HLA-DR4-transgenic mice display more robust antigen presentation by B cells than males, and increased IL-4 and IL-13 production, which further promotes B cell responses (202, 204). In contrast, HLA-DR4 males produce more anti-inflammatory IL-10 and have higher numbers of Tregs and Bregs (204). Treg frequencies decrease following castration of these male mice supporting immunosuppressive effects of androgens (204).

Oestrogen supplementation to mice with collage-induced arthritis limits disease development and severity by reducing IFN-γ and TNF-α production, as well as by shifting autoantibody isotype from IgG2a to IgG1 (205–207). In addition, estrogen decreases Th17 cells in the joints during established arthritis by promoting their migration to lymph nodes, *via* ER-α-mediated upregulation of CCR6 on Th17 cells and CCL20 in lymph nodes (206). Androgens also suppress RA, in part due to inhibition of NF-κB and subsequent reduction in IL-6 (208, 209).

The T cell co-stimulatory molecule CD2 is involved in T cell activation, as well as Th17 and Treg differentiation (202). CD2 is associated with RA and is expressed at higher levels in the RA synovium and healthy PBMCs of women than men (210). In addition, it has recently been demonstrated that a polymorphic ER-binding site regulates expression of surrounding genes, including CD2, in a sex-specific manner, resulting in sexually dimorphic T cell responses (202).

Altogether, these studies implicate a significant role for genetic factors, such as HLA type, along with sex hormones for the sex-specific lymphocyte differences in RA.

### Myasthenia gravis

In MG pathogenic autoantibodies cause neuromuscular junction transmission failure and muscle weakness, which can be life-threatening (19). Interestingly, the age of onset is a critical determinant of sex bias in MG. In female predominant early-onset MG (EOMG; <50 years), there is lymphoid follicular hyperplasia (LFH) and germinal centers containing numerous B cell clones within the thymus (150, 211). Thymocytes and circulating T cells from patients with MG express more ER-α and -β than controls (66). The upregulation of ERs has been shown to be a result of increased pro-inflammatory cytokines IFN-γ, IL-1 and TNFα (66), that likely enhance reactivity to estrogens, and promote B cell responses. Furthermore, estrogen exposure in experimental autoimmune MG has been shown to enhance Th1 responses, and production of IgG2a and IgG2b autoantibodies, as well as exacerbate disease (212).

In male predominant late-onset MG (LOMG), the thymus is atrophied, and autoimmunity is thought to result from a lack of AIRE, along with a loss of muscle-like myoid cells, resulting in a failure of negative T cell selection to muscle-specific antibodies (213–215). Thymic involution is known to progress at a faster rate in males than females, and in LOMG there is a decrease in naive cell export from the thymus, with a trend to lower export in males, perhaps in part explaining the male bias to disease (213, 216).

Genetic studies have also demonstrated sex-specific HLA alleles to associate with the different subtypes of MG. HLA-B*08 and HLA-DR3 associate with EOMG, with a stronger association in females (217, 218). In contrast, LOMG is associated with HLA-B7 and/or HLA-DR2 alleles in males but not females (218, 219).

## Factors that influence sex bias in lymphocytes

Understanding and acknowledging sex bias needs to be at the forefront of future immunological research. There are several additional factors detailed below that should be taken into consideration, due to their potential influence on sex differences in lymphocytes.

### Hormonal influences

Hormonal effects vary between autoimmune diseases, as detailed in the previous sections. As such, differences in exposures to hormonal therapies between the sexes also needs to be considered in the context of specific autoimmune diseases. For instance, high-dose estrogen supplementation in female MS patients reduces IFN-γ expression by PBMCs, and results in an improvement in disease (185). In contrast, in SLE, use of hormonal contraceptives may increase risk of developing disease, and hormone replacement therapy use is linked to an increase in mild-moderate disease flares (220, 221).

Male patients with MS treated with testosterone displayed reduced CD4 T cells, increased NK cells, reduced IL-2 production from PBMCs, and reduced brain atrophy (222, 223). Gonadotrophin-releasing hormone (GnRH) antagonists, used in prostate cancer treatment, reduce testosterone levels. GnRH antagonists block thymocyte proliferative responses and reduce numbers of CD4 T cells, Tregs and B cells (224–226). GnRH agonists have also been shown to exert sexually dimorphic actions in an SLE mouse model, with worsening of disease seen only in females, potentially due to gender differences in the expression of G proteins on immune cells (227). Worsening of MS in females has also been reported with their use (228–230).

### Ageing

Immunosenescence that occurs with age results in both a reduced functional capacity of the immune system to protect against infection as well as enhanced immune-mediated inflammation, commonly known an “inflammaging” (231, 232). This decline in immune function occurs earlier in men than women, but the rate of decline is faster in women upon onset, paralleling changes in sex hormone levels, and making the sex difference in lymphocytes more divergent (233–236). In addition to hormonal changes, sex-based differences in the aging of lymphocytes have been attributed to increased skewing of XCI in females (237), differences in mitochondrial dysfunction (238), telomere shortening (239), chromatin accessibility (233) and demethylation (240).

As T cells age, there is a sex difference in the upregulation of genes related to inflammaging. Older females display higher proinflammatory responses and weaker T cell mediated defenses compared to age-matched males (236, 238). There is an increase in memory/activated T cells, and an increased CD4:CD8 T cell ratio, with a greater rate of increase in women (52, 241–243). Women also demonstrate a later but stronger rise in IL-6 than men (244). IFN-γ transiently increases in the early postmenopausal period in women (237, 245, 246), before falling to its lowest level in late menopause (247). In contrast, IFN-γ levels have been seen to either decline or not change with age in healthy men (232, 246). In older men with inflammatory or autoimmune diseases however, IFN-γ and IL-17 production by T cells is higher than younger men, which increases with disease severity, a pattern not seen in older women with the same diseases (232).

Although females have more antibody-producing plasma cells than males, this trend has been shown to weaken with age (235). Further, a subset of dysfunctional B cells known as age-associated B cells (ABCs), are associated with autoantibody production and are expanded in elderly females (248–250). It is proposed that overexpression of TLR7 in females may lead to accumulation of this autoantibody-producing B cell subset (251). In patients with SLE, IgD-CD27- double negative (DN) B cells, which are similar to ABCs, are expanded and display hyper-responsiveness to TLR7 (252, 253).

Overall, sex-specific changes in lymphocytes with age are complex and not yet fully understood, however, provide some explanation as to why autoimmune disease susceptibility differs between men and women with age.

### Interaction with the target organ

Sex differences have been demonstrated in lymphocyte interaction with target organs of various autoimmune diseases (189, 242, 243, 254, 255). These are influenced by sex differences in target organ structure and cell death pathways, as well as sex hormone interaction with the target tissue. Despite increased prevalence of autoimmune disease in women, in many conditions males appear to be prone to greater target organ damage. This is demonstrated by increased apoptosis of thymocytes and thymic atrophy in male rodents than in females given lipopolysaccharide, which binds to TLR4 on macrophages to trigger a pro-inflammatory cascade promoting a Th1 response (256, 257).

In MS, male sex is a risk factor for poor outcome and increased brain atrophy (183). This is likely to be, in part, due to sex differences in miRNA expression within the brain (258, 259), differences in blood-brain barrier permeability (254), and the ability of estrogen to exhibit neuroprotective effects (260). Studies in hypoxic brain injury and stroke have demonstrated differences in Treg responses, and their effects upon cerebral infiltration and brain injury between men and women, resulting in Treg-mediated neuroprotection in females but increased neurodegeneration in males (255, 261).

In contrast to MS, female patients with RA on TNF-α inhibitors display lower remission rates in comparison to males (34, 262). This may be explained by the higher expression of CD2 in the synovium of females; *in vitro* deletion of CD2 has been shown to reduce the damaging effects of TNF-α on synovial cells (210, 263). In addition, there is thought to be a male-biased benefit of anti-TNF-α-driven reduction in the peripheral conversion of steroid precursors to estrogen in synovial tissues in RA (264–266), levels of which correlate with inflammation (149). Finally, synovial secretion of IL-6 and IL-8 positively correlate with the expression of ER-α and ER-β, but not AR (267), suggesting that estrogen signaling might contribute to synovial inflammation in females.

### Differences in the innate immune system

Differences in the innate immune system perpetuate the more robust immune responses seen in females. Neutrophils and macrophages have higher phagocytic activity in females than males (268), and female antigen presenting cells are more efficient at presenting peptides (269). Testosterone reduces expression of TLR4, reduces synthesis of TNF, and increases BAFF, IL-10, IL-12 and TGF-β production from macrophages (88, 129, 270, 271). Low dose estrogen activates dendritic cells (DCs) (272, 273), whereas (272, 273)high dose estrogen increases DC tolerance, reduces proinflammatory cytokine production and increases IL-10 and TGF-β mRNA expression (274). Sex differences in the complement system have also been demonstrated, including increased activity of the alternative pathway in males (275, 276), contrasting to a greater dependence on the lectin pathway in females (277). Female mice have been shown to display reduced susceptibility to complement-mediated damage, possibly due to differences in terminal pathway components (278). It is therefore unclear why complement-mediated diseases are more common in women, but this may contribute towards the increased male-susceptibility to target organ damage.

### Gut microbiome

Mouse studies have shown that differences in the gut microbiome, with influence from sex hormones, contribute to sex differences in the immune response. This has been well summarized in a recent review (279). Conflicting data exists in human studies, in which it is harder to account for the multiple confounding factors. However, sex differences have been shown in the gut microbiome in numerous studies, with influence from sex hormones (280–282). Thus, its influence upon autoimmune disease and mechanisms to modulate this requires further investigation.

### Other factors

In addition to genetic and hormonal factors, sex differences in exposure and response to environmental factors including sociological differences, psychological stress, diet, obesity, vitamin D, smoking, and viral infections may also impact upon the sex differences in lymphocytes (283–288). It is likely that these factors contribute towards the increasing incidence seen in autoimmune diseases by 3 to 9% each year (1).

Women are more likely to develop cross-reactive T and B cells to foreign stimuli such as cigarette smoking, viruses, and dietary peptides, which can act as triggers for autoimmune disease (289–291). Females are also more likely to be obese, with higher amounts of leptin produced by their adipocytes. This promotes pro-inflammatory Th1 responses and inhibits Tregs (292–294). In patients with MS, the immunomodulatory effects of vitamin D were found to be greater in females than in males including inhibition of T cell proliferation, reduction in IFN-γ and IL-17, and an increase in Tregs and IL-10-secreting cells (295). Additionally, an independent study also showed that vitamin D promotes Treg differentiation and can ameliorate EAE in an estrogen-dependent manner (296).

## Conclusion

The influence of sex chromosomes, modulated by direct and indirect effects of sex hormones, contributes to sex differences in lymphocyte populations and their functions. Although the contribution of these differences to specific autoimmune diseases remains to be fully understood, it is apparent that they contribute to the disparities in disease susceptibility, severity, and response to treatment between men and women. It is therefore important to acknowledge these differences in lymphocytes when evaluating treatment strategies, or disease biomarkers, as a step towards personalized medicine for individuals with autoimmune diseases. Future research aimed to further elaborate the sex bias in the immune response could provide novel tailored treatment strategies for improved management of autoimmune diseases.

## Author contributions

KD and MM have been involved in the drafting of the manuscript and approved the final version.

## Funding

MM is funded by the Academy of Medical Sciences Springboard Award (SBF006\1165), Asthma & Lung UK (RP22F\6) and University of Manchester Presidential Fellowship. KD is undertaking a PhD funded by the Manchester Myasthenia Gravis Research Fund (NorthCare Charity Fund CS2041), with additional grants from Myaware and the Neuromuscular Study Group, and a salary from the Northern Care Alliance NHS Foundation Trust.

## Acknowledgments

We thank P. A. Blair and B. Potts for their comments on the manuscript.

## Conflict of interest

The authors declare that the research was conducted in the absence of any commercial or financial relationships that could be construed as a potential conflict of interest.

## Publisher’s note

All claims expressed in this article are solely those of the authors and do not necessarily represent those of their affiliated organizations, or those of the publisher, the editors and the reviewers. Any product that may be evaluated in this article, or claim that may be made by its manufacturer, is not guaranteed or endorsed by the publisher.
